# Microbiology and Nitrogen Cycle in the Benthic Sediments of a Glacial Oligotrophic Deep Andean Lake as Analog of Ancient Martian Lake-Beds

**DOI:** 10.3389/fmicb.2019.00929

**Published:** 2019-05-03

**Authors:** Victor Parro, Fernando Puente-Sánchez, Nathalie A. Cabrol, Ignacio Gallardo-Carreño, Mercedes Moreno-Paz, Yolanda Blanco, Miriam García-Villadangos, Cristian Tambley, Virginie C. Tilot, Cody Thompson, Eric Smith, Pablo Sobrón, Cecilia S. Demergasso, Alex Echeverría-Vega, Miguel Ángel Fernández-Martínez, Lyle G. Whyte, Alberto G. Fairén

**Affiliations:** ^1^Centro de Astrobiología (CSIC-INTA), Madrid, Spain; ^2^SETI Institute, Carl Sagan Center, Mountain View, CA, United States; ^3^NASA Ames Research Center, Mountain View, CA, United States; ^4^Campoalto Operaciones SpA, Santiago, Chile; ^5^Instituto Español de Oceanografía (IEO), Málaga, Spain; ^6^Muséum National d’Histoire Naturelle, Paris, France; ^7^School of Environmental Sciences, University of Guelph, Guelph, ON, Canada; ^8^Centro de Biotecnología, Universidad Católica del Norte, Antofagasta, Chile; ^9^Vicerrectoría de Investigación y Postgrado, Universidad Católica del Maule, Talca, Chile; ^10^Department of Natural Resource Sciences, McGill University, Montreal, QC, Canada; ^11^Department of Astronomy, Cornell University, Ithaca, NY, United States

**Keywords:** microbiology of deep benthic habitats, Andean lakes, deglaciation, ammonia oxidation, nitrification-denitrification, benthic sediments, planetary exploration, planetary lakes

## Abstract

Potential benthic habitats of early Mars lakes, probably oligotrophic, could range from hydrothermal to cold sediments. Dynamic processes in the water column (such as turbidity or UV penetration) as well as in the benthic bed (temperature gradients, turbation, or sedimentation rate) contribute to supply nutrients to a potential microbial ecosystem. High altitude, oligotrophic, and deep Andean lakes with active deglaciation processes and recent or past volcanic activity are natural models to assess the feasibility of life in other planetary lake/ocean environments and to develop technology for their exploration. We sampled the benthic sediments (down to 269 m depth) of the oligotrophic lake Laguna Negra (Central Andes, Chile) to investigate its ecosystem through geochemical, biomarker profiling, and molecular ecology studies. The chemistry of the benthic water was similar to the rest of the water column, except for variable amounts of ammonium (up to 2.8 ppm) and nitrate (up to 0.13 ppm). A life detector chip with a 300-antibody microarray revealed the presence of biomass in the form of exopolysaccharides and other microbial markers associated to several phylogenetic groups and potential microaerobic and anaerobic metabolisms such as nitrate reduction. DNA analyses showed that 27% of the Archaea sequences corresponded to a group of ammonia-oxidizing archaea (AOA) similar (97%) to *Nitrosopumilus* spp. and *Nitrosoarchaeum* spp. (Thaumarchaeota), and 4% of Bacteria sequences to nitrite-oxidizing bacteria from the *Nitrospira* genus, suggesting a coupling between ammonia and nitrite oxidation. Mesocosm experiments with the specific AOA inhibitor 2-Phenyl-4,4,5,5-tetramethylimidazoline-1-oxyl 3-oxide (PTIO) demonstrated an AOA-associated ammonia oxidation activity with the simultaneous accumulation of nitrate and sulfate. The results showed a rich benthic microbial community dominated by microaerobic and anaerobic metabolisms thriving under aphotic, low temperature (4°C), and relatively high pressure, that might be a suitable terrestrial analog of other planetary settings.

## Introduction

A cold and wet ancient Mars around 3.8 Gyr (Fairén, 2010) together with high-obliquity cycles allowed snow precipitation, glacier formation as well as all the companion processes as deglaciation and lake formation (Fairén, 2017). In fact, current NASA’s MSL (Mars Science Laboratory) mission is taking place on an ancient lake floor in crater Gale (Hurowitz et al., 2017). In an ancient Gale lacustrine scenario (Hurowitz et al., 2017) a subglacial lake might have alternated with deglaciation periods (Fairén, 2017) that provided sediments and nutrients to the system to support anaerobic microbial populations.

In terrestrial analogs of wet and cold Early Mars, specific environmental conditions, such as periods of upstream permafrost freezing and thawing, or enlargement of the catchment area, result in an extra nutrient supply to glacial lakes (Rogora et al., 2008; Salerno et al., 2016). If these conditions persist, they can lead nutrient-poor lakes (oligotrophic) toward nutrient-rich (eutrophic) stages (Lyons et al., 2006), that can end in complete eutrophication where blooming of certain species change the evolution of the lake (Li et al., 2016). These natural feeding processes might have occurred very often along the Earth history as well as in other planetary bodies such as ancient Mars, where hydrothermal, meteoritic impacts, and glacial environment coexisted (Neukum et al., 2004). Quick desiccation or freezing processes of those water bodies might have trapped concentrated traces of potential microbial biomarkers along the geological record, as it has been described on Earth (Hays et al., 2017).

Benthic nitrogen cycle plays a key role for life development in aquatic environments, as well as for understanding lake trophic status (Gettel et al., 2013). This is especially relevant in the nutrient enrichment of oligotrophic lakes, which are commonly N-limited (Elser et al., 2009), sensitive to atmospheric nitrogen deposition (Lepori and Keck, 2012), and to the increased water catchment (Antoniades et al., 2011). Nitrogen limitation may be more severe in the deep benthic zones where light is insufficient for phototroph primary producers, especially nitrogen fixing cyanobacteria (Gettel et al., 2013). The availability of fixed nitrogen from geological sources or decomposing organic matter, for example in the form of ammonium (NH_4_^+^) or ammonia (NH_3_), may be further constrained by its mineralization through nitrification activities (Gettel et al., 2013). Ammonia (NH_3_) oxidation to nitrite (NO_2_^-^) is the first reaction of the nitrification process in nature and it is catalyzed by ubiquitous bacterial (ammonia oxidizing bacteria, AOB) or archaeal (ammonia oxidizing archaea, AOA) groups (Monteiro et al., 2014). The second reaction is the oxidation of nitrite to nitrate (NO_3_^-^)by nitrite-oxidizing bacteria (NOB), although it has been recently reported that a single bacterium strain is capable to perform both reactions (Daims et al., 2015). On Mars, the most likely inorganic source of nitrogen is nitrate (NO_3_^-^), which has been detected in Gale Crater at concentrations ranging from 100 to 300 ppm in aeolian samples, and from 70 to 1100 ppm in sediments (Stern et al., 2015). However, it is still unclear whether a primitive N cycle ever developed on Mars. For example, processes such as the post-depositional behavior of nitrates and the recycling of oxidized N back into the atmosphere, including nitrate reduction by Fe(II) in aqueous environments (Price et al., 2018), are still unknown to occur on Mars.

Oligotrophic lakes appear as suitable environments for the development of archaeal populations capable of ammonia oxidation to nitrite (Jenkins and Kemp, 1984). It is well documented that low ammonium concentrations would select for AOA over AOB because of their higher affinity for ammonium (Martens-Habbena et al., 2009). In recent years, several studies have shown the diversity of nitrifying prokaryotes, its distribution and its role on nitrogen cycle both in marine and terrestrial environments, including the water column of freshwater lakes and high altitude glacial lakes (Bartrons et al., 2010; Auguet et al., 2012). However, very little is known about the microbial processes on the nitrogen cycle in the deep benthic zone sediments of these lakes. During the Planetary Lake Lander (PLL) project, whose main focus was the development of technology for planetary lake exploration (Pedersen et al., 2015), we investigated and sampled the water column and the deep benthic sediments of Laguna Negra, a glacial and oligotrophic lake of 12 km^2^ and located in the Central Andes of Chile, with the goal of collecting data on its benthic ecosystem, and to assess its capacity to buffer the whole-system response to nutrient addition as a consequence of deglaciation. In a previous work (Parro et al., 2018), we reported the presence of a rich microbial community in the shallow sediments of the lake shore that may account for a variety of anaerobic metabolisms (e.g., sulfate reduction, methanogenesis, or anammox-anaerobic ammonia oxidation). In another report (Echeverría-Vega et al., 2018), we showed that limnological features and microbial diversity of the water column down to 20 m were consistent with the oligotrophic and photic character of the upper water column. Here, we expand the study of the Laguna Negra by reporting the microbial diversity and evidences of the main operating metabolisms at the deepest aphotic benthic sediments. The ecosystem is driven by microaerobic/anaerobic metabolisms where nitrification/denitrification processes seem to play a central role. Finally, we extrapolate this ecological model to early Mars lake scenarios that were subjected to similar hydrological processes, with particular emphasis on an hypothetical full nitrogen cycle.

## Materials and Methods

### Sampling the Benthic Zone of the Laguna Negra

Sampling from the Laguna Negra benthic zone were carried during two sampling campaigns, one in April 2015 and another one in May 2015. The equipment for sampling consisted of (i) Two zodiac boats equipped with sonar, GPS, and electrical motors; (ii) a wood hoist system was set up between the two Zodiacs, and a rope rolling system for a 300 m × 8 mm rope; (iii) a special *gopro* case and underwater flashlights, together with an aluminum lander for cameras and lights, and; (iv) an Ekman grab (May) for sample collection sediments down to approximately 10 cm. A 1.8 L sample (S1) was collected on the 5th of April 2015, at 264 m depth, at coordinates S 33°38′39.6″, W 70°07′42.9″. One month later (5th of May) a 2.0 L (including water and sediments) of a second sample (S2) was collected 541 m far from the first one and from 269 m depth, at coordinates S 33°38′42.9″, W 70°07′22.26″. Each sample was distributed into 3–4 500 mL bottles, and immediately kept in a cooler, stored refrigerated, and one bottle shipped to Madrid (Spain) for analysis. Samples were stored in a cold room (4°C) until used for LDChip and DNA extraction (2 months later) and Mesocosms experiment (9 months later). Temperature at the lake bed was 4°C and was measured with a YSI 6600 multi-parameter probe onboard the lake lander platform.

### Geochemical Analysis

In previous work we reported the geochemistry of the Laguna Negra waters down to 20 m depth from samples collected and filtered on site (Echeverría-Vega et al., 2018). In the present work, the samples were stored at 4°C and geochemical analysis was carried out 2 months after sampling. To determine the anion content (inorganic ones such as Cl^-^, Br^-^, NO_3_^-^, NO_2_^-^, PO_4_^2-^, SO_4_^2-^, and small organic ones such as acetate, formate, propionate, tartrate, oxalate) in the water around the samples, 2 g of wet sediment were centrifuged at 2000 *g* for 10 min to separate the interstitial water (IW) from the coarse solid material. Then, the supernatant was directly analyzed by ion chromatography using a Metrohm 861 Advanced Compact Ion Chromatographer IC (Metrohm AG, Herisau, Switzerland), set up to detect all the anions indicated above in a single run, as described in Parro et al. (2011a). The ion chromatograph was calibrated for measuring, in a single run, the presence of several inorganic and organic anions. For each anion, 6 different concentration within the range shown below were used to make the calibration curves: Fluoride (2–0.08 ppm), Chloride (10–0.4 ppm), Nitrite (5–0.2 ppm), Bromide (2–0.08 ppm), Nitrate (50–2 ppm), Sulfate (200–8 ppm), Acetate (5–0.2 ppm), Propionate (2–0.08 ppm), Formate (2–0.08 ppm), Phosphate (2–0.08 ppm), Tartrate (2–0.08 ppm), and Oxalate (2–0.08 ppm). Under these conditions the limit of detection is in the range of 0.005–0.010 ppm in the run sample. Ammonium concentration was determined with a colorimetric method with the Reflectoquant^®^ 20–180 mg L^-1^ Ammonium Test kit (Merk) following the provider’s instructions.

### Antibody Microarrays: Printing LDChip and Fluorescent Sandwich Immunoassay

The Life Detector Chip (LDChip) is an antibody microarray-based biosensor specifically developed for planetary exploration and environmental monitoring (Rivas et al., 2008; Parro et al., 2011a). The LDChip used in this work contained over 300 antibodies including antibodies reported previously (Parro et al., 2011a, 2018) and new ones (Supplementary Table S1). The new set of rabbit polyclonal antibodies were produced, as previously reported (Rivas et al., 2008), against exopolysaccharide (EPS) material and whole cellular lysates from several strains of psychrophilic microorganisms (Supplementary Table S1) isolated from High Canadian Arctic (Prof. Lyle Whyte, McGill University collection) and perchlorate reducing bacteria (from Prof. John Coates, Berkeley University). The strains belong to the genera: *Brevundimonas*, *Micrococcus*, *Frigoribacterium*, *Frondihabitans*, *Polaromonas*, *Sporosarcina*, *Rhodococcus*, *Polaromonas*, *Paenibacillus*, *Bacillus*, *Tumebacillus*, *Dechlorobacter*, *Azospira*, *Arcobacter* (see Supplementary Table S1 for details). Antibody purification, titration, printing onto microscope slides, antibody fluorescence labeling and multiplex microarray immunoassays were carried out as described in Rivas et al. (2008), Parro et al. (2011a),and Blanco et al. (2017).

One of the advantages of LDChip is the capability of interrogating for hundreds of microbes or molecular markers in a single sandwich assay immunoassay by using very little amount of sample with minimal processing. LDChip is the core sensor of the instrument SOLID (Signs of Life Detector) developed for planetary exploration (Parro et al., 2011b). Although in the work we follow a manual procedure, we always emulate the SOLID sample processing and analysis procedures in a permanent task of demonstrating the strengths of the system for doing science in planetary exploration. Therefore, only 0.5 g of sediments of each collected sample were suspended in 2 ml of TBSTRR buffer (0.4 M Tris-HCl pH 8, 0.3 M NaCl, 0.1% Tween 20) and sonicated using a manual ultrasonicator (Dr. Hielscher 50 W DRH-UP50H sonicator, Hielscher Ultrasonics, Berlin, Germany) for 3 × 1 min cycles, with 30 s pauses on ice. Then, samples were filtered through 8-μm nitrocellulose filters to remove sand and coarse material. 50 μl of each filtrate were used as multianalyte-containing sample for fluorescence sandwich microarray immunoassays (FSMI) as described in previous works (Parro et al., 2011a, 2018; Blanco et al., 2017). As a blank control, only buffer was incubated with the chip instead of sample. LDChip microarray images were analyzed and quantified by Genepix Pro Software (Molecular Devices, Sunnyvale, CA, United States) as previously reported (Parro et al., 2011a;Rivas et al., 2011).

### DNA Extraction, Cloning, Sequencing and Analysis

Total DNA was extracted from two aliquots of 0.5 g of wet weight from all sediment samples by using the MoBio Power-Soil DNA extraction kit (MoBIO laboratories), according to the manufacturer’s instructions. The prokaryotic V3-V4 hypervariable region of the 16S rRNA gene (Bacteria and Archaea) was PCR amplified with primers Bakt_341F (5′CCTACGGGNGGCWGCAG3′) and Bakt_805R (5′GACTACHVGGGTATCTAATCC3′) (Herlemann et al., 2011) as described (Klindworth et al., 2013), as well as the eukaryotic intergenic ITS1-4 region of 18S rRNA gene using primers CTTGGTCATTTAGAGGAAGTAA as forward and CGYCAATTNMTTTNAGT as reverse (Ghannoum et al., 2010), and then sequenced following the Illumina Miseq system (Lyfesequencing, Valencia, Spain). In addition, Archaeal 16S rRNA gene was PCR amplified using the primers 20F 5′TTCCGGTTGATCCYGCCRG3′ as forward, and U1392R 5′ACGGGCGGTGTGTRC3′ as reverse using 20 ng of metagenomic DNA, 200 μM of each of the four deoxynucleoside triphosphates, 400 nM of each primer, 2.5 U of FastStart HiFi Polymerase, and the appropriate buffer with MgCl_2_ supplied by the manufacturer (Roche, Mannheim, Germany) using the following steps: denaturation at 95°C for 4 min, 30 cycles of denaturation at 95°C for 30 s, annealing at 48°C for 45 s, and elongation at 72°C for 90 s, and a final extension at 72°C for 10 min. The PCR amplicons were purified with Qiagen PCR purification kit columns (Qiagen, CA, United States), cloned into plasmid vector pTOPO-TA (Invitrogen), and sequenced by Sanger following ABI PRISM 3710 capillary sequencer system (ThermoFisher).

The resulting paired-end libraries were subjected to assembly (bacterial and eukaryotic libraries) and quality-filtering (all libraries) using the *moira.py* script (Puente-Sanchez et al., 2016) with a truncation length of 250 bp. For the archaeal sequences, truncation length was of 200 bp and the analysis was restricted to the forward reads, as the reverse reads were found to contain a high proportion of low quality bases. Also, non-archaeal reads were removed after using mothur v1.36.1 (Schloss et al., 2009) to classify all reads against the silva.v119 reference database provided by its authors. After quality-filtering, bacterial and archaeal reads were analyzed with mothur as described previously (Kozich et al., 2013). Briefly, sequences were aligned to mothur’s version of the SILVA alignment (Quast et al., 2013), scanned for putative chimeras with UCHIME (Edgar et al., 2011), and clustered into Operational Taxonomic Units (OTUs) using a 3% distance cutoff. The resulting OTUs were classified with mothur v1.36.1 (Schloss et al., 2009) using the *classify.otu* command and the silva.v119 reference database provided by its authors. The eukaryotic reads were analyzed in a similar way, but a multiple sequence alignment was constructed with MUSCLE (Edgar, 2004) instead of aligning the reads to the SILVA reference alignment. Eukaryotic OTUs were taxonomically classified using SINA (Pruesse et al., 2012).

The near full-length archaeal 16S rRNA amplicons obtained following Sanger sequencing (see above) were aligned with SINA (Pruesse et al., 2012) and inserted into the SILVA SSU Ref NR 99 reference tree using the parsimony algorithm included in the *arb* software suite (Ludwig et al., 2004). The query sequences and their closest matches were selected to generate a simplified phylogenetic tree. TreeGraph2 (Stöver and Müller, 2010) was used for final figure formatting. Sequences for archaeal clones have been deposited in the NCBI Genbank database Acc. No: KY693649-KY693659. Raw MiSeq sequences have been deposited in the NCBI SRA database, and can be accessed from BioSample Acc. No: SAMN10919703 and SAMN10919695.

### Ammonium Oxidizing Activity Assay

Two separate mesocosm experiments were carried out 9 months after sampling and storage at 4°C to test ammonia oxidizing activity and nitrate and sulfate evolution. First, to test whether ammonium could be transformed and mineralized via nitrification, we suspended 6 g (w/w) of S2 sample sediment collected in May 2015 into 150 mL with sterile, Ion Chromatography grade water. Equal amounts (50 mL) were split into three identical cultures in 250 mL flasks. Two of them only contained the diluted sediment sample and the other one was supplemented with 1000 μg mL^-1^ of ampicillin to avoid most of the bacterial growth without affecting archaea. The samples were incubated in the dark at 4°C with mild agitation at 140 rpm. 5 mL aliquots were withdrawn for ion chromatography assay at 0, 12, 24, 48, 72, 96, and 240 h of incubation. Nitrate and sulfate concentrations were determined by ion chromatography as follows: 5 ml aliquots were centrifuged at 4000×*g* for 6 min at 4°C and then the supernatant filtered through 0.22 μm pore size filter, and injected into the Ion Chromatograph as described in Parro et al. (2011a). A second experiment was done by using the same amounts as above and by splitting into three identical 50 mL cultures in 250 mL flasks. This time, one of them was maintained with no additional reagents as control, another one was supplemented with 1000 μg mL^-1^ of ampicillin, and the third one with 100 μM of 2-Phenyl-4,4,5,5-tetramethylimidazoline-1-oxyl 3-oxide (PTIO) (Sigma-Aldrich), a specific ammonia oxidizing activity inhibitor in ammonia oxidizing archaea (AOA) (Martens-Habbena et al., 2015; Sauder et al., 2016). Ammonium, nitrate and sulfate concentration were measured as indicated above but including two extra samplings, at 6 and 120 h. Although we determined the ammonium concentration (as indicated above) in all the sub-cultures samples, only those from the controls were valid because the presence of PTIO and ampicillin strongly affected ammonia measurement system and provided erroneous values.

## Results

### Exploring the Benthic Zone of a Deep Andean Lake

**FIGURE 1:**
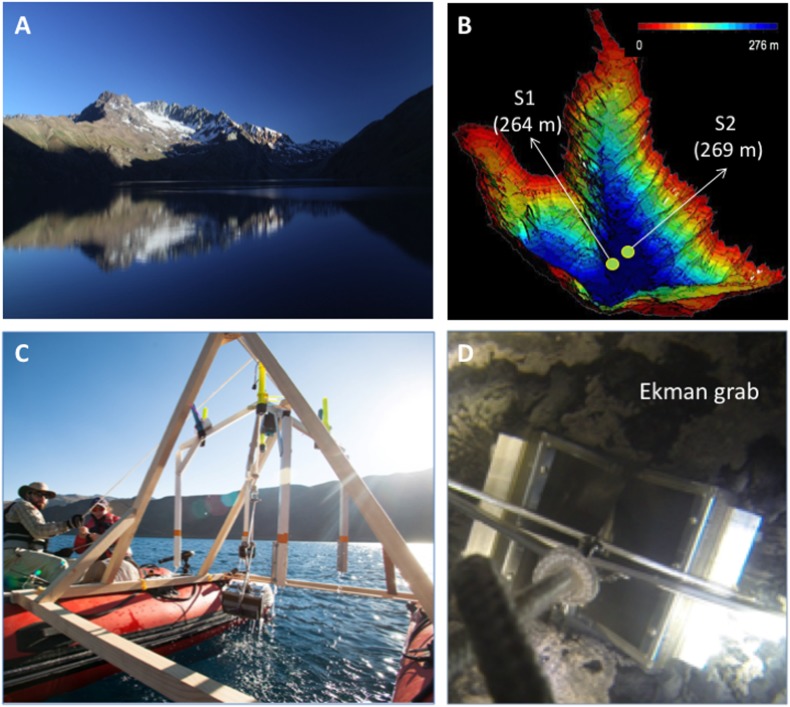
Environmental assessment and sampling of the benthic sediments of Laguna Negra. **(A)** Laguna Negra, in the Región Metropolitana (Chile) with the Echaurren glacier in the background. **(B)** Bathymetric map of Laguna Negra showing the sampling sites and depths. **(C)** Sampling system using two zodiac boats allowed the collection of sediment from benthic sites shown in **B**. **(D)** Sampling with an Ekman drag system at the moment of contact with the lakebed atS2 (269 m depth).

Laguna Negra is an oligotrophic lake located on the south slope of the Echaurren glacier watershed in the Central Andes of Chile (33.65S/70.13W). It is part of a complex of freshwater resources of the Santiago area, which also includes Lo Encañado and El Yeso lake and dam, respectively. Laguna Negra is located around 2,700 m above sea level with an extension of 6.1 × 1.7 km, and a maximum depth of 276 m (Pille, 2013). Two sediment samples were collected in 2015, S1 at 264 m depth and S2 at 269 m (see section “Materials and Methods” and Figure 1) for rapid biomarker profiling with LDChip and molecular ecology studies. The average temperature measured *in situ* through the lake bed was 4°C.

Ion chromatography analysis of the interstitial water for determining the concentration of small-size organic and main inorganic anions showed the oligotrophic character of the Laguna Negra waters: acetate, propionate, tartrate, oxalate, bromide, nitrite, and phosphate were below detection limit of our system (see Materials and Methods). Only fluctuating concentration of formate (24 ppb), chloride (64 ppb), nitrate (129 ppb), and sulfate (3,182 ppb) were measured in S2 sample, as well as up to 2.8 ppm (0.15 μM) ammonium. Only formate (23 ppb) and sulfate (123 ppb) was measured in S1.

### Microbial Mass and Biomarkers at the Benthic Zone of Laguna Negra

**FIGURE 2:**
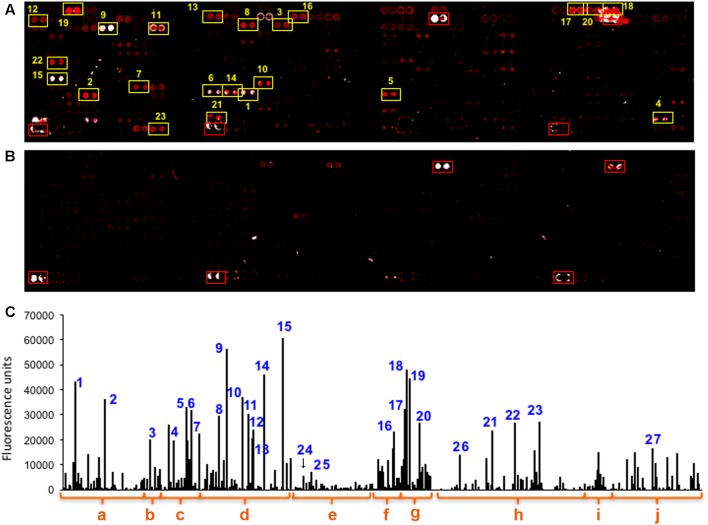
The multiplex immunoassay LDChip detected abundant microbial immunoreactive polymeric material in the benthic zone of Laguna Negra. **(A)** LDChip showing fluorescent spots (duplicate pattern) indicating positive immunodetection after the analysis of one of the samples obtained at 265 m depth. **(B)** Blank control where only the buffer solution was incubated instead of the sample. **(C)** The fluorescence intensity of all the spots in image **A** was quantified and plotted after subtraction of the background and the nonspecific antibody fluorescence in the blank control image. The bars with numbers indicate the spots selected in image **A**, and correspond to antibodies raised against different groups of microbes or molecular markers: **(a,b)** antibodies to cells and EPS from iron-sulfur rich environments, and hydrothermal and cold environments; **(c)** iron and sulfur oxidizers acidophiles such as *Leptospirillum* spp (4), *Acidithiobacillus* spp (5,6), or *Halothiobacillus* spp. (7); **(d)** psychrophilic microorganisms from different phylogeny as *Polaromonas* spp. (8), *Paenibacillus* spp. (9), *Shewanella gelidimarina* EPS (10), EPS from environmental isolate lhp2530 (11), EPS from *Planococcus* spp (12), the Actinobacteria *Arthrobacter* spp. (13), *Psychroserpens burtonensis* EPS (14), *Colwellia psychrerythraea* EPS (15); **(e)** a group of mesophile bacteria with different metabolisms, such as the sulfate reducer *Desulfovibrio vulgaris* (24), the methanotroph *Methylomicrobium* sp. (25), several Firmicutes and Actinobacteria that did not showed significative immunodetections; **(f)** Cyanobacteria, such as *Leptolyngbya* spp, *Nostoc*, *Microcystis* spp., *Aphanizomenon ovalisporum* (16); **(g)** a group classified as perchlorate reducing bacteria but with very versatile metabolisms comprising *Arcobacter* spp. cells (17) and their EPS (18); *Azospira* spp EPS (19), *Dechlorobacter hydrogenophilus* EPS (20); **(h)** key proteins and peptides from different metabolic processes as GlnB (21) from nitrogen metabolism, a K^+^ transporter (22), NifD and NifH (23) for nitrogen fixation, or ApsA (26) for dissimilatory sulfate reduction; **(i)** Cell extracts from other environments; **(j)** and complex polymeric substances as humic acids (27).

Planetary exploration requires of quick and reliable *in situ* analytical methods. We first investigated microbial biomass content as well as biodiversity by using an antibody microarray immunosensor (LDChip, see section “Materials and Methods”). Although the analyses were performed in the laboratory, we emulated the procedures usually done in the field with low amount of sample and minimal processing. The samples consisted of fine and loose sediments that easily formed homogeneous suspensions in water. Up to 0.5 g (wet weight) of S1 and S2 samples were processed to obtain a crude extract and analyzed by fluorescence sandwich microarray immunoassay (FSMI) with LDChip. The fluorescent images of the microarray and the immunogram obtained after fluorescence quantification (Figure 2) identified positive immunodetections with several antibodies in S2 (similar results were obtained with S1. Not shown). Overall, the LDChip reported diverse and rich microbial markers, and indicated that some metabolic processes were occurring in the sample. Several antibodies in the LDChip detected EPS material and cellular remains that may be similar to EPS from acidic biofilms and polar environments, as inferred from the antibodies that showed positive signals. Several positive immunodetections corresponded to antibodies produced against psychrophilic microorganisms such as *Polaromonas* sp., *Paenibacillus* sp., *Shewanella gelidimarina*, *Planococcus* sp. EPS, *Arthrobacter* sp. (Actinobacteria), *Psychroserpens burtonensis* EPS, or *Colwellia psychrerythraea* EPS, in agreement with the low temperature of the environment (4°C). Other antibodies produced against iron-sulfur oxidizers and reducers such as *Leptospirillum* spp., *Acidithiobacillus* spp. and *Halothiobacillus* spp. showed positive detection. Biomarkers from Cyanobacteria were also detected such as those from *Aphanizomenon ovalisporum*, *Microcystis aeruginosa*, *Tolypothrix distorta*, and *Anabaena* sp. Additionally, positive immunodetections corresponded to the diazotrophic (nitrogen fixer) bacteria *Arcobacter* sp. and *Azospira suillum* which, together with *Dechlorobacter hydrogenophilus*, are also capable of dissimilatory perchlorate reduction under anaerobic conditions. In agreement with the presence of diazotrophic strains, some immunodetections corresponded to proteins and peptides involved in nitrogen metabolism such as GlnB (a regulatory protein of nitrogen metabolism), and specifically the nitrogen fixation NifD and NifH (key protein components of the Mo-Fe nitrogenase), a K^+^ transporter (important, among other functions, in keeping internal cellular homeostasis), or an important protein in dissimilatory sulfate reduction as it is ApsA (adenylylsulfate reductase alpha subunit). This is in agreement with the immunodetection (although with low signal) of *Desulfovibrio vulgaris*, the same genus of the strain (*D. desulfuricans*) from which the peptide sequence was used to produce the anti-ApsA antibody (Supplementary Table S1). Although we expected some immunodetections with antibodies to methanogens such as *Methanosarcina mazei* or Methanobacterium formicicum, and other sulfate reducers such as *Desulfosporosinus meridiei*, no significant signals were obtained with the corresponding antibodies.

### Sequencing the rRNA Gene Confirmed a Rich Microbial Community

**FIGURE 3:**
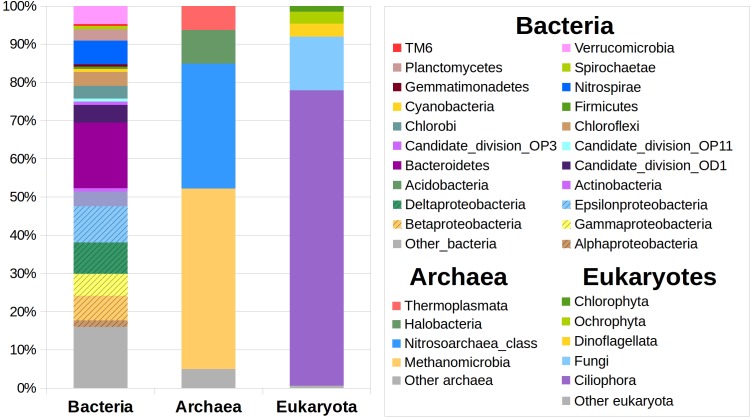
Microbial diversity in the benthic zone of Laguna Negra. High-throughput sequencing of the 16S rRNA gene of prokaryotes and 18S gene of eukaryotes (see Materials and Methods) allowed to identify the main phylogenetic groups of Bacteria, Archaea, and Eukaryota, and to estimate the relative proportions of taxa within each group. See text for discussion.

Total DNA was extracted from the S1 and S2 samples for high throughput bacterial, archaeal, and eukaryotic rRNA gene sequencing and analysis (Figure 3). An initial analysis of the DNA sequences showed a very similar phylogenetic affiliation patterns in both samples (not shown). Therefore, we treated them as a single pool of sequences and considered it as an average representative sample from the benthic zone of the lake. Out of all DNA sequence reads, we clustered 8830 from Bacteria, 1907 from Archaea, and 17022 from Eukarya, which accounted for 2283 (Bacteria), 117 (Archaea), and 5875 (Eukarya) different OTUs at 97% similarity. These data produced diversity indexes (Shannon/invSimpson) values of 6.36/91.77, 2.20/3.97, and 6.70/44.83 for Bacteria, Archaea, and Eukarya, respectively. Only 6103 (36%) of the ITS sequences could be taxonomicaly classified; this problem was absent from the 16S (bacterial and archaeal) datasets. The most abundant bacterial DNA sequences corresponded to the sulfur oxidizing *Sulfuricurvum* genus (9% of all the bacterial sequences), Bacteroidetes from the vadinHA17 group (9%), the Candidate division OD1 (5%), or the nitrite oxidizing *Nitrospira* genus (4%; raises to 7% when considering the whole *Nitrospiraceae* family). The Archaea were dominated by the methanogenic *Methanoregula* (41%) and *Methanosaeta* (6%), and the *Nitrosoarchaeum* (Thaumarchaeota phylum) group (27%), and *Halobacteriales* from the Deep_Sea_Hydrothermal_Vent_Gp_6 group (8%). Most of the eukaryotic sequences corresponded to Ciliates (77%), ten times less fungi from the LKM-11(7.05%) and LKM-15 (3.44%) clades found in Antarctic lakes, and dinoflagellates (2%). The eukaryotes provide biomass to the system, and can produce specific compounds such as steroid hormones which can be degraded by microorganisms (see below). No sequences were retrieved from sponges or tubeworms.

For a more accurate phylogenetic adscription of the *Nitrosoarchaeum* related group of sequences, larger 16S rRNA gene fragments were amplified by PCR with specific primers for Archaea, cloned and sequenced from S2 DNA. Nine out of fourteen retrieved sequences corresponded to marine ammonia-oxidizing archaea (AOA) with more than 97% similarity to *Nitrosopumilus maritimus* and “Candidatus *Nitrosopumilus koreensis*” (Supplementary Figure S1). These microorganisms are usually found in ammonia-oxidizing marine environments as well as associated with the microbiota of sponges and tubeworms animals. Additionally, some sequences corresponded with >98% similarity to methanogenic archaea, such as *Methanosaeta* spp. and *Methanoregula* spp., in agreement with the high-throughput sequencing results.

### Ammonia Oxidizing Activity in the Benthic Sediments of Laguna Negra

**FIGURE 4:**
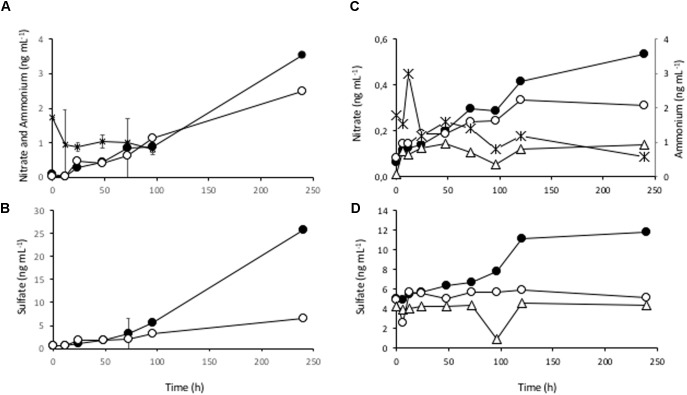
Ammonium oxidizing activity in the benthic sediments at Laguna Negra. Mesocosm incubation at 4°C for several days and sampling for measuring the ammonium consumption and nitrate and sulfate accumulation along time course (0, 6, 12, 24, 48, 72, 96, 120, and 240h). **(A)** Average concentrations of two replicate samples showing the ammonium consumption (asterisks) and nitrate evolution in mesocosm samples without inhibitors or control samples (filled circles). The nitrate concentration in the presence of ampicillin (open circles) was also measured from a single flask. **(B)** Evolution of sulfate in the same samples as in **A** without inhibitors (closed circles) and with ampicillin (open circles). **(C)** Nitrate evolution in another experiment without inhibitors (closed circles), with ampicillin (open circles), and with 100 μM PTIO (open triangles). Again, ammonium consumption was measured (asterisks). **(D)** Sulfate evolution from the same samples as in **C**, without inhibitors (closed circles), ampicillin (open circles), and PTIO (open triangles).

To demonstrate ammonium oxidation and nitrification activities suggested by the DNA sequence data, several aliquots of the S2 sample were incubated at 4°C (the water temperature in the lake) and the concentrations of nitrate, sulfate and ammonium were monitored over time (Figure 4 and Supplementary Figures S2, S3). The results showed a drop in the ammonium concentration while nitrate and sulfate accumulated in the medium, indicating that ammonium oxidation was indeed taking place. In order to definitely assign the ammonium oxidizing activity to AOA strains, one aliquot of the sample was treated with the nitrogen free radical scavenger 2-phenyl-4,4,5,5,- tetramethylimidazoline-1-oxyl-3-oxide (PTIO), a specific inhibitor of the archaeal ammonium oxidation (Martens-Habbena et al., 2015). In parallel, another aliquot was supplemented with ampicillin, an antibiotic that inhibits only bacterial growth, with no effects on the archaeal communities. In both samples, with ampicillin and with PTIO, the nitrate measured in the medium was lower than in the control without inhibitors (Figure 4) after several days. In the presence of ampicillin nitrate still accumulated and increased along time (always below the control), while with PTIO its concentration did not significantly change after 10 days of incubation (Figure 4). The concentration of sulfate in the medium, however, did not change significantly from the initial conditions and remained constant in both treatments, indicating that any biological process involved in sulfate production (sulfur compounds oxidation) was inhibited either with PTIO alone, or ampicillin alone (see discussion below). Therefore, we conclude that the mesocosm experiments revealed a relevant ammonia oxidizing activity coupled to nitrification processes which, in turn, may be coupled to the oxidation of sulfur compounds.

## Discussion

### Ammonia-Oxidizing Driven Metabolisms in Deep Benthic Sediments of Laguna Negra

Sampling, sample storage, and shipping conditions in remote environments is always a major issue. We decided keeping all the samples refrigerated (4°C) to avoid additional alterations due to unpredictable freeze and melting during shipping. Instead, we cannot rule out any other alteration of the geochemistry and microbiology of the sample, but at least they were maintained under the most similar conditions as possible to the natural environment, that is, low temperature and minimal oxygen. It can be expected that highly altered samples would allow the growth of filamentous parasitic and saprophytic fungi and yeasts feeding from weak and dead microbiota as well as other degraded biological material (Crawford et al., 1990). In our case, no yeast were detected and only <0.1% of DNA sequences were related to filamentous fungi such as *Penicillium* spp. and *Aspergillus* spp. that could indicate any type of sample contamination or alteration. Instead of that, we identified DNA sequences attributed to *Cryptomycota* clades LKM11 and LKM15 (Lara et al., 2010), highly abundant in ice-covered oligotrophic lakes in the Antarctic Dry Valleys (Rojas-Jiménez et al., 2017) (Figure 3).

**FIGURE 5:**
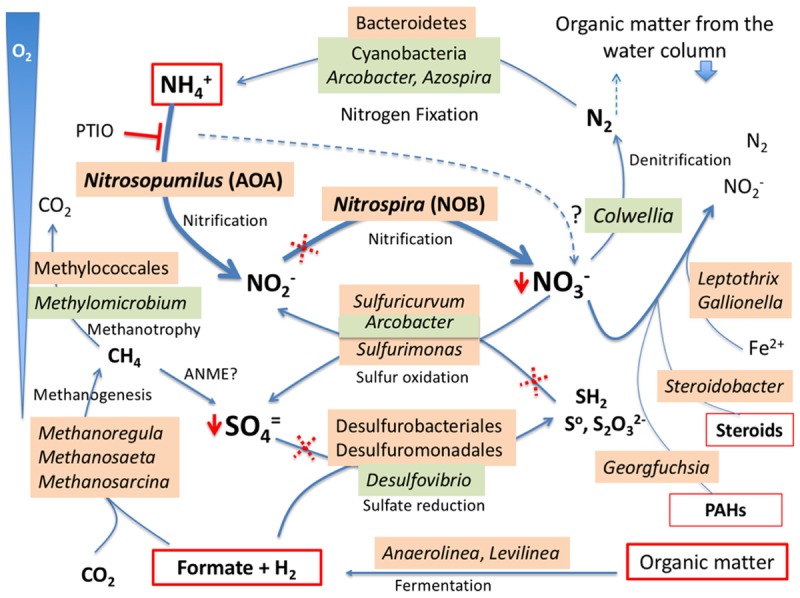
Anaerobic and microaerobic metabolisms in the benthic microbial community of Laguna Negra. Under microaerobic conditions, the ammonium from decomposing organic matter fuels the *Nitrosopomilus*-like ammonia oxidizing archaea (AOA) coupled to nitrite oxidizing bacteria (NOB) (nitrifying) from *Nitrospira* genus to produce nitrate. In turn, nitrate is a good electron acceptor in most anaerobic niches for the oxidation of sulfur compounds by sulfur oxidizing bacteria (SOB) or steroid compounds from benthic microfauna. Alternatively, nitrate may follow a complete denitrification process to N_2_ which, in turn, and considering the oligotrophy of the waters, can be fixed again by Cyanobacteria, *Azospira*, or *Arcobacter* species, closing the whole nitrogen cycle. The organic matter fermentation renders small organic acids as acetate and formate that are used as reducing power for methanogenesis or/and sulfate reduction in the anaerobic niches. Dotted red crosses indicate the steps where ampicillin should inhibit the bacterial growth during the mesocosm experiments (Figure 4). The reaction inhibited by PTIO is also indicated (**T**). Both PTIO and ampicillin lower the nitrate and sulfate accumulation (red arrowhead). Phylogenetic groups shadowed in orange were detected with DNA, and those in green with LDChip. The question (?) mark on the dashed line suggests that any AOA present in the medium could be able to complete the full ammonia oxidation to nitrification (see text for further discussion).

The geochemical, immunological, and DNA sequence analyses revealed a rich microbial community in the benthic sediments, as well as suggested potentially viable microaerobic and anaerobic metabolisms driving this ecosystem (Figure 5). Significant amounts of ammonium (0.15 μM) may be the substrate for AOA, as suggested by the relatively high number of DNA sequences of *Nitrosopumilus* spp. Ammonia oxidation produces nitrite which in turn is used by nitrite oxidizing bacteria (NOB) as *Nitrospira* spp, already detected at relevant proportions in the DNA pool (see above). The spatiotemporal co-occurrence of these two types of prokaryotes suggests a syntrophic relationship, where the AOA oxidize ammonium to nitrite and the NOB oxidize nitrite to nitrate. A similar co-occurrence has been described in soil and marine environments (Santoro et al., 2010; Xia et al., 2011), and relatively high proportion of DNA sequences from Thaumarchaeota and Nitrospirae phyla were reported in the sediments of two freshwater lake in the Tibetan Plateau (Zhang et al., 2015). Herein, we report 16S rDNA sequences at the genus level and metabolic evidences of the association of AOA and NOB, both at relatively high proportions, in samples from benthic sediments (269 m of water column) of a freshwater, oligotrophic, and glacial Andean lake. Nitrate may be the electron acceptor in other metabolisms such as the oxidation of reduced sulfur compounds or organic molecules, or can be fully reduced to N_2_ by denitrifying bacteria such as some species of *Colwellia*. In addition, other bacteria may be involved in denitrification such as *Leptothrix* spp. and *Gallionella* spp. [both oxidize Fe(II) coupled to nitrate], or *Steroidobacter* spp. *and Georgfuchsia* spp. (that oxidize steroids produced by microfauna and polyaromatic hydrocarbons- PAHs- from humic substances, respectively, coupled to Nitrate reduction). In turn, N_2_ could be released to the water column and the atmosphere or be fixed again by diazotrophic (nitrogen fixing) bacteria that we also identified by DNA sequencing and by immunological assays (*Azospira* sp., *Arcobacter* sp., or some cyanobacterial species as *Nostoc* or *Aphanizomenon* spp). Moreover, antibodies to nitrogen fixation proteins (NifH and NifD) showed positive immunodetections as well (Figure 2).

The mesocosm incubation experiments in the laboratory showed ammonium oxidation with accumulation of nitrate and sulfate at 4°C, suggesting a direct connection between these processes (Figure 5). One limitation of the experiment is that it was done under aerobic conditions, different from the original environment. However, because under these conditions anaerobic processes are inhibited, we could infer the metabolic connections between ammonia oxidation and nitrate and sulfate reduction through the accumulation of this compounds. In a certain way we were using oxygen to block anaerobic processes. In the natural setting, it is expected that operative anaerobic sulfate and nitrate reduction metabolisms would immediately use these compounds and avoid their accumulation. The addition of ampicillin did not inhibit completely the accumulation of nitrate, which was, however, fully stopped in the presence of 100 μM PTIO. As PTIO is a nitric oxide scavenger that preferentially inhibits the ammonia oxidation by AOA isolates and natural communities (Martens-Habbena et al., 2015), this result suggested that not all NOB strains were sensitive to ampicillin. Another hypothesis is that an unknown AOA strain in the Laguna Negra sample might be able to complete alone the ammonium oxidation to nitrate. This last case would be equivalent to that reported for an AOB strain of the genus *Nitrospira*, which is capable of performing complete nitrification (Daims et al., 2015; Van Kessel et al., 2015). Whatever the case, further work is necessary with fresh sediment samples to fully verify this metabolic scheme.

Our model for the benthos of Laguna Negra (Figure 5), where the highest ammonia concentration recorded was 0.15 μM, is consistent with other *in situ* studies with microbial isolates and mesocosms showing that AOA populations grow with low ammonia concentrations, in contrast to AOB that require significantly higher ammonia concentrations to initiate growth (Verhamme et al., 2011). Indeed, some AOA species as *N. maritimus* did not tolerate ammonia at concentrations significantly above 1 mM (Martens-Habbena et al., 2009). Therefore, the AOA reported herein seem to be the main drivers of ammonia oxidation in the sediments of this oligotrophic lake, and would outcompete the AOB which, as the inhibition experiment with PTIO indicated, are not able to perform complete nitrification to accumulate nitrate.

The fact that sulfate accumulation stopped from the beginning of the experiment in the presence of ampicillin could be explained by growth inhibition of the sulfur oxidizing bacteria (SOB) responsible for its production (Figure 4). The accumulation of both nitrate and sulfate stopped in the presence of PTIO, suggesting that sulfate accumulation might be coupled to nitrate concentration, and a consequence of the activity of sulfur oxidizing bacteria (e.g., *Sulfuricurvum*, *Arcobacter*, or *Sulfurimonas* genera detected) that used nitrate as electron acceptor (Figure 5). In an oxygen limited environment, Park et al. (2008) reported mutual benefits between SOB and AOA, and proposed that a rapid oxygen consumption by the SOB to oxidize reduced sulfur compounds depleted the oxygen, and allowed AOA to proliferate and outcompete AOB. This is consistent with the prevalence of AOA instead of AOB in oligotrophic and suboxic environments such as the oceanic oxygen minimum zones (Francis et al., 2005).

The presence of small organic acids such as formate, mostly from microbial fermentation processes, may act as electron donors to support growth of sulfate reducing bacteria (SRB) and/or acetoclastic methanogenic archaea. We retrieved DNA sequences from Desulfurobacteriales and Desulfuromonadales orders (Deltaproteobacteria), which include sulfate reducing species, and also sequences from methanogens such as *Methanosaeta* and *Methanoregula*. It has been reported that methanogens and SRB compete for reduced substrates, such as acetate, formate and H_2_ (Lovley and Klug, 1983). In our samples, acetate was negligible and formate concentration was low, indicating that either the rate of fermentation was very slow due to the low temperature, or that these substrates were quickly consumed by methanogens or SRB. Either way, the coexistence of several methanogens genera suggested that a variety of substrates might be available. This setting is similar to the one we reported for the shallow sediments of the Lo Encañado lake near Laguna Negra (Parro et al., 2018), where we detected several methanogens coexisting with SRB in an environment with low acetate and sulfate concentration.

Neither DNA sequences nor the immunodetection signals are indicative of actual metabolisms at the sampling time. However, they are consistent indicators of the metabolic potential of the sample and, by extension, the natural environment where they came from. For example, the detection of some DNA sequences attributed to Cyanobacteria as well as clear immunological reactions with LDChip may be due to the inputs of dead cells from the water column, as they were previously detected at different depths by DNA sequencing and by immunoassay (Echeverría-Vega et al., 2018; Parro et al., 2018). Alternatively, cyanobacterial strains could, in fact, have been adapted to heterotrophy under low oxygen and the absence of light in these sediments, similarly, as it has been described in other environments (Moezelaar et al., 1996; Miyatake et al., 2013). Or they might be able to use the H_2_ produced by some fermenting bacteria (e.g., *Anaerolinea* and *Levilinea* genera) as reducing power, as it has been suggested recently for viable cyanobacteria in the deep continental subsurface (Puente-Sánchez et al., 2018).

### Terrestrial Glacial Lakes as Analogs of Lakes in the Cold and Wet Early Mars

**FIGURE 6:**
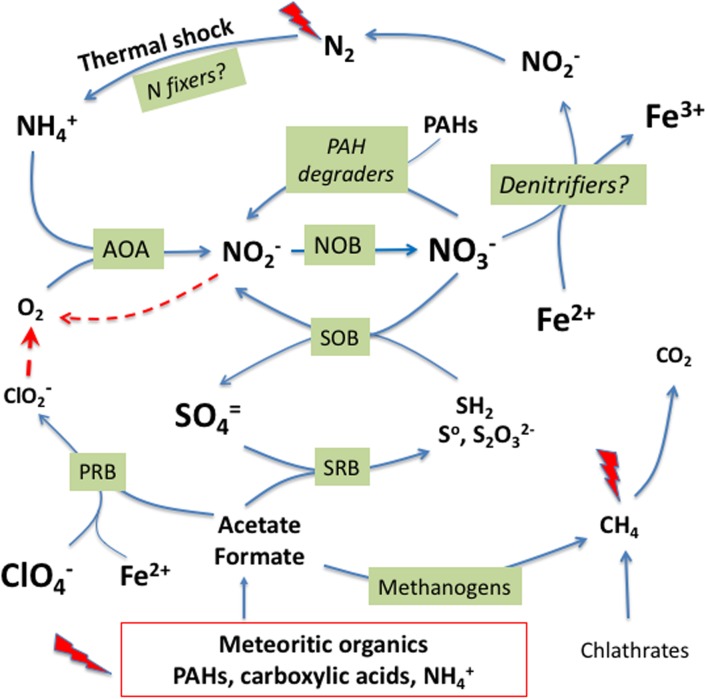
A hypothetical microbially mediated nitrogen cycle in the lake bed sediments of ancient lakes on Mars. Meteoritic organic inputs, photochemical reactions and thermal shock on atmospheric N_2_ would produce ammonium to feed the lake through runoff waters from deglatiation. In the presence of minimal concentration of oxygen from nitrite and chlorite dismutation, ammonium would be oxidized to nitrate by potential AOA and/or NOB. Iron oxidizers could use nitrate as electron acceptor to initiate a denitrification step that would end in N_2_ to close the cycle. On the other hand, carboxylic acids can fuel anaerobic metabolisms as sulfate and perchlorate reduction, methanogenesis, as well as heterotrophic, and fermentation processes (not shown). See text for details.

The cycling of N in Laguna Negra could represent an analog for understanding the potential for nitrogen cycling on early Mars and its availability for potential microbiota (Figure 6). We hypothesize that a potential martian microbiology could have contributed to close the martian nitrogen cycle in the deepest sediments of ancient lakes as the one at crater Gale. The sediments would provide salts and oxidants such as sulfate (Milliken et al., 2014) and iron oxides (Hurowitz et al., 2017), and nutrients such as a range of organics from meteorite impacts (PAHs and small organic acids as acetate and formate) and even ammonia (Benner et al., 2000). In fact, a diversity of organic compounds have been detected in Gale sediments formed in ancient lacustrine environments (Freissinet et al., 2015; Eigenbrode et al., 2018). Microorganisms equivalent to the terrestrial AOA and NOB would have contributed to generate nitrate that, together with the nitrate produced by abiotic processes as thermal shocks (Summers and Khare, 2007), would be an electron acceptor for Fe(II) oxidizers (Price et al., 2018) that contribute to denitrification to N_2_. Alternatively, nitrate and other oxidants such as Fe^3+^ or perchlorate (ClO_4_^-^), could all be electron acceptors for other metabolisms such as sulfur oxidation (SOB) and degradation of organic matter (PAHs consumers). The minimal amount of oxygen (O_2_) required for ammonia oxidation could be supplied by chlorite and/or nitrite dismutation (Ettwig et al., 2012). Nitrate, could also be fully reduced to dinitrogen (N_2_) by denitrifying microbes through coupling to ferric iron oxidation. Abiotic processes as photochemical reactions and thermal shocking or potential nitrogen fixing microbes could produce again nitrate or ammonia, closing the cycle. In those lakes with residual hydrothermal activity at the floor (Schulze-Makuch et al., 2007; Fairén et al., 2010; Ruff and Farmer, 2016), reduced compounds as CH_4_, SH_2_, or NH_3_ might have provided energy to methanotrophs and reducing power to sulfur and ammonia oxidizers.

One of the main factors affecting the criteria for the selection of future landing site for Mars exploration missions is the knowledge of their ancient hydrogeological record. Our work can contribute to understand the type of life that might have inhabited those lakes as well as the type of potential biosignatures, including preserved organic molecules, organic structures (e.g., cells), mineral indications of life, and isotopic markers. Our work demonstrated once more the usefulness of the LDChip, the core sensor of SOLID instrument (Parro et al., 2011b) as key analytical system for *in situ* life detection in planetary exploration (McKay et al., 2013).

## Author Contributions

VP, NC, and AF conceived the case study. IG-C, MM-P, FP-S, MF-M, VT, YB, MG-V, AE-V, CD, and LW performed experimental research (analytical techniques, cultures, and DNA analysis). CrT, ES, PS, and CoT performed sampling campaign and *in situ* measurements. VP and AF wrote the manuscript. All the authors read and approved the final manuscript.

## Conflict of Interest Statement

The authors declare that the research was conducted in the absence of any commercial or financial relationships that could be construed as a potential conflict of interest.
