# Supplementary material for: Microbiology and Nitrogen Cycle in the Benthic Sediments of a Glacial Oligotrophic Deep Andean Lake as Analog of Ancient Martian Lake-Beds
Source: Front Microbiol. 2019 May 3;10:929. doi: 10.3389/fmicb.2019.00929 (PMC6509559; doi:10.3389/fmicb.2019.00929)
Supplement: Supplementary file 2 [file Data_Sheet_2.pdf]

Supplementary Material

Microbiology and Nitrogen Cycle in the Benthic Sediments of a Glacial Oligotrophic Deep Andean Lake as Analog of Ancient Martian Lake-Beds

Victor Parro[1]*, Fernando Puente-Sánchez[1], Nathalie A. Cabrol[2,3], Ignacio Gallardo-Carreño[1], Mercedes Moreno-Paz[1], Yolanda Blanco[1], Miriam García-Villadangos[1], Cristian Tambley[4], Virginie C. Tilot[5,6], Cody Thompson[7], Eric Smith[2], Pablo Sobrón[2], Cecilia S. Demergasso[8], Alex Echeverría-Vega[9], Miguel A. Fernández-Martínez[1], Lyle G. Whyte[10], and Alberto G. Fairén[1,11].

***CORRESPONDECE: Victor Parro**

Department of Molecular Evolution, Centro de Astrobiología (CAB, INTA-CSIC), Carretera de Ajalvir km 4, Torrejón de Ardoz, 28850, Madrid, Spain.

Phone:+34-915201071; Fax: :+34-915201074; email: parrogv@cab.inta-csic.es

Running Title: Microbiology at the benthic zone of an Andean lake

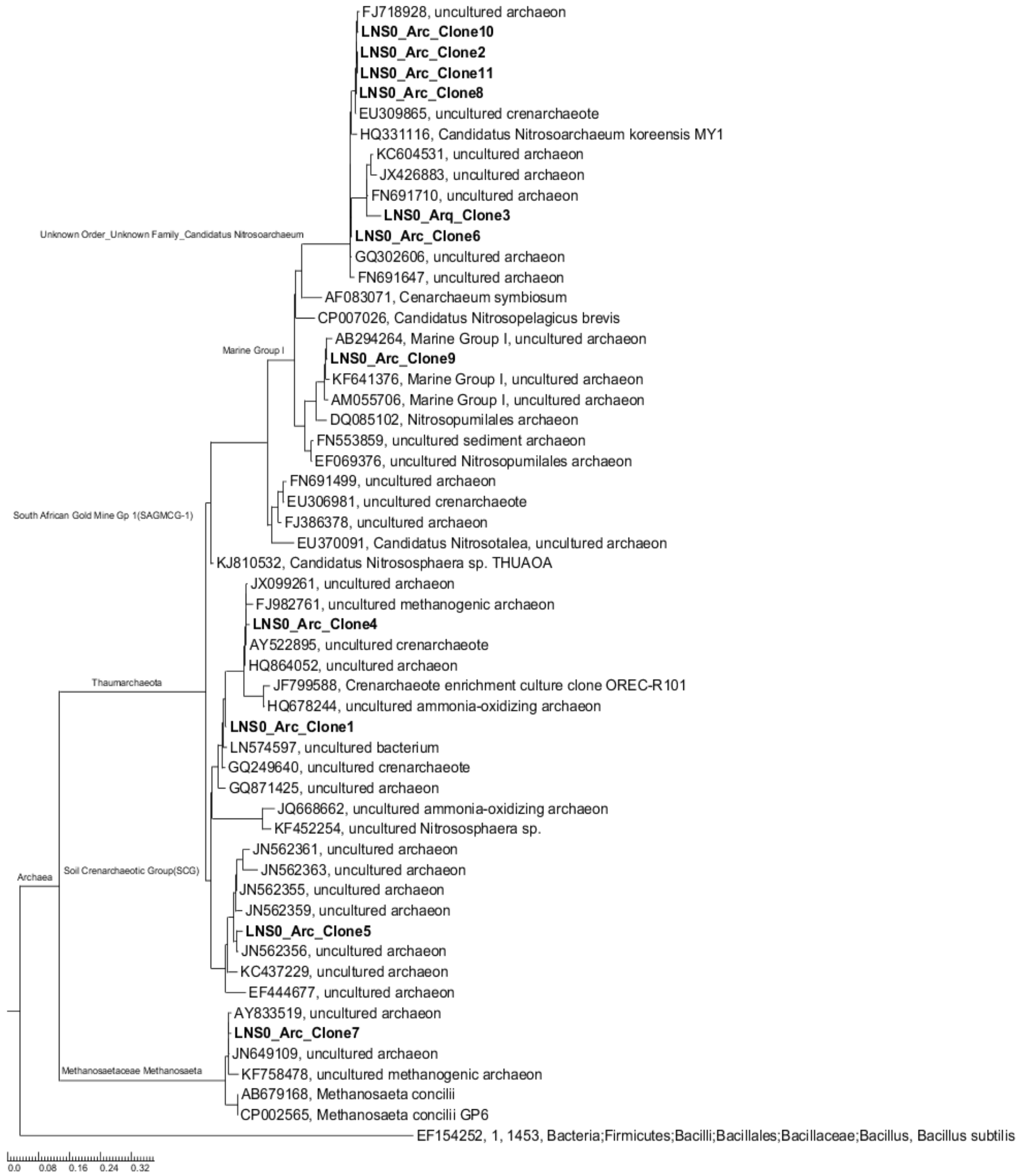

Figure S1. DNA sequences attributed to ammonia oxidizing archaea (AOA) from the benthic sediments of Laguna Negra obtained by PCR amplification, cloning, and sequencing. A phylogenetic tree of the sequences shows high similarity with other ammonia oxidizing archaea (AOA) such as *Nitrosoarchaeum* spp. and *Nitrosopumilus* spp. or highly related environmental isolates classified as part of the Thaumarchaeota phylum.

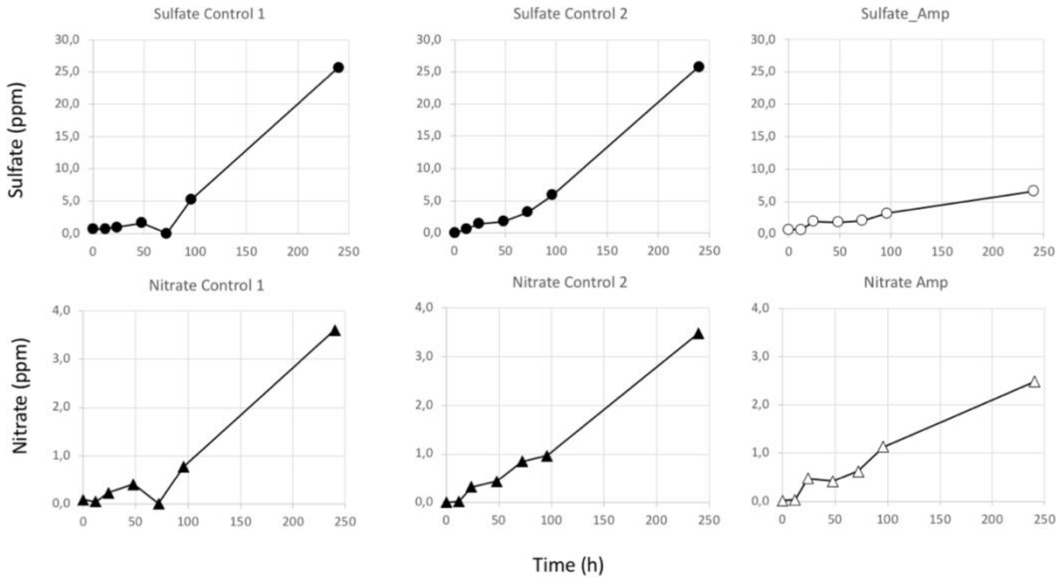

Figure S2. Individual replicates sulfate and nitrate concentrations of the experiment shown in Fig. 4 A,B. Control 1 and 2 refer to two replicate samples incubated without addition of inhibitors. The replicate values were very similar, that is why no error bar are visible in Fig. 4 A,B except in one point. Amp, ampicillin, only one sample.

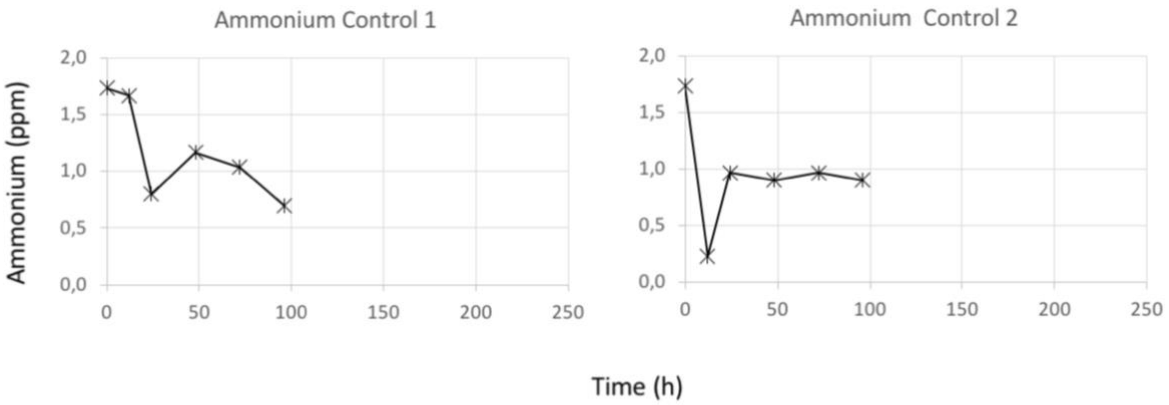

Figure S3. Individual replicates ammonium concentrations of the experiment shown in Fig. 4 A,B. Control 1 and 2 refer to two replicate samples incubated without addition of inhibitors. No ammonium was detected in the Amp (ampicillin) supplemented sample, perhaps as a consequence of any interference with the measuring method.
